# Remote limb ischemic postconditioning inhibits microglia pyroptosis by modulating HGF after acute ischemia stroke

**DOI:** 10.1002/btm2.10590

**Published:** 2023-08-19

**Authors:** Lu Yu, Zuohui Zhang, Hao Chen, Miao Wang, Wenqi Mao, Jinxia Hu, Dandan Zuo, Bingchen Lv, Weifeng Wu, Suhua Qi, Guiyun Cui

**Affiliations:** ^1^ Department of Neurology The Affiliated Hospital of Xuzhou Medical University, Xuzhou Medical University Xuzhou China; ^2^ School of Medical Technology, Xuzhou Key Laboratory of Laboratory Diagnostics Xuzhou Medical University Xuzhou China

**Keywords:** acute ischemic stroke, high throughput screening, microglial, pyroptosis, remote limb ischemic postconditioning

## Abstract

The repetitive inflation–deflation of a blood pressure cuff on a limb is known as remote limb ischemic postconditioning (RIPostC). It prevents brain damage induced by acute ischemia stroke (AIS). Pyroptosis, executed by the pore‐forming protein gasdermin D (GSDMD), is a type of regulated cell death triggered by proinflammatory signals. It contributes to the pathogenesis of ischemic brain injury. However, the effects of RIPostC on pyroptosis following AIS remain largely unknown. In our study, linear correlation analysis confirmed that serum GSDMD levels in AIS patients upon admission were positively correlated with NIHSS scores. RIPostC treatment significantly reduced GSDMD level compared with patients without RIPostC at 3 days post‐treatment. Besides, middle cerebral artery occlusion (MCAO) surgery was performed on C57BL/6 male mice and RIPostC was induced immediately after MCAO. We found that RIPostC suppressed the activation of NLRP3 inflammasome to reduce the maturation of GSDMD, leading to decreased pyroptosis in microglia after AIS. Hepatocyte growth factor (HGF) was identified using the high throughput screening. Importantly, HGF siRNA, exogenous HGF, and ISG15 siRNA were used to reveal that HGF/ISG15 is a possible mechanism of RIPostC regulation in vivo and in vitro.

**Translational Impact Statement**

Remote limb ischemic postconditioning (RIPostC), a noninvasive protection strategy, could alleviate brain damage induced by acute ischemia stroke (AIS). Pyroptosis contributes to the pathogenesis of ischemic brain injury. Our study demonstrates for the first time that RIPostC plays an antipyroptosis role after AIS in vivo, vitro, and clinical trials, which involves HGF/ISG15. This finding provides evidence for the clinical application of RIPostC in AIS. Moreover, we propose that HGF has the potential to be utilized as a target biomarker in RIPostC treatment.

## 1 INTRODUCTION

Stroke is a leading cause of physical disability globally and affects ~17 million people yearly.^1^ In patients with acute ischemic stroke, besides timely and effective reperfusion, adjunct therapies are also necessary to decrease infarct sizes and ameliorate the clinical outcomes.^2^, ^3^ More recently, remote limb ischemic postconditioning (RIPostC) is a noninvasive protection strategy that induces ischemic conditions on a remote organ (limbs) after cerebral ischemia, intending to prevent brain injury after AIS.^4^, ^5^, ^6^ Even though multiple studies have validated the neuroprotective effects of RIPostC, including modulating Microglia/Macrophage Polarization and shifting circulating monocytes to the CCR2+ proinflammatory subset,^7^, ^8^, ^9^ its underlying mechanisms have not been fully elucidated.

As the resident immune cells of the central nervous system, microglial cells are the key cellular mediators involved in acute neuroinflammatory responses.^10^ Pyroptosis is a proinflammatory form of programmed cell death. It relies on the activity of cytosolic GSDMD and is driven by inflammasomes.^11^ Microglia pyroptosis has been implicated in the pathogenesis of multiple CNS diseases, including SCI, traumatic brain injury, and ischemic stroke.^12^, ^13^, ^14^ NLRP3 inflammasome is a key mediator that plays an important role in pyroptosis; it is reported to be the main member expressed in microglia.^15^ Upon activation, the NLRP3 inflammasome converts precursor caspase‐1 into cleaved caspase‐1, which then cleaves precursors interleukin (IL)‐1β and IL‐18 into biologically active mature proinflammatory cytokines and cleaves GSDMD to trigger pyroptosis.^16^, ^17^ Therefore, it could be potentially useful to evaluate whether the immunosuppressive mechanism of RIPostC is involved in the regulation of microglia pyroptosis after AIS.

Hepatocyte growth factor (HGF) is a powerful pleiotropic cytokine. It induces angiogenesis, mitosis, and tissue regeneration, as well as exerts anti‐apoptotic and anti‐inflammatory effects in various organs.^18^, ^19^ HGF administration might prevent disruption of the blood–brain barrier (BBB), attenuate inflammatory responses, and bring neuroprotective effects after cerebral ischemia.^20^ Recently, studies have demonstrated that HGF could shift M1 macrophages toward an M2‐like phenotype and inhibit septic endothelial pyroptosis.^21^, ^22^ Furthermore, HGF could increase the expression levels of interferon‐stimulated gene 15 (ISG15).^23^ After ISG15 removal in human microglia, the expression levels of IL‐1β were significantly upregulated, but the underlying mechanism is unclear.^24^

We hypothesized that RIPostC could reduce NLRP3‐mediated microglia pyroptosis, subsequently reducing neuroinflammation after AIS. In the current study, we first confirmed the role of GSDMD and HGF in AIS patients and mice after the RIPostC application. Then, in vivo and in vitro experiments were used to investigate whether the mechanism of RIPostC regulating microglia pyroptosis is associated with the HGF/ISG15 axis.

## 2 RESULTS

### 2.1 RIPostC improved acute ischemic stroke outcomes after AIS

To test the hypothesis that RIPostC improves AIS outcomes, we enrolled 42 AIS patients within 3 days after ischemic onset and divided them into two groups: with RIPostC group and without RIPostC group. The clinical characteristics of the patients are shown in Table 1. NIHSS score evaluation at 3 and 7 days as well as mRS score at 90 days after treatment was conducted in both two groups. The results indicated that RIPostC treatment led to statistically significant reductions in 90 days mRS score compared with the without RIPostC group, while 3 and 7 days NIHSS score after treatment with no significant difference between the two groups (Table 2).

**TABLE 1:** Clinical characteristics of patient samples.

	Total	Without RIPostC	With RIPostC	*p*
Patients (*n*)	42	21	21	
Sex				
Male (*n*)	26	14	12	0.53
Female (*n*)	16	7	9	
Ages (years)	—	70 [59,76]	75 [66,81]	0.186
Admission NIHSS score	—	18 [10,24]	19 [12,21.5]	0.591
Hypertension (*n*)	29	13	16	0.726
Diabetes (*n*)	15	8	7	1
Smoking history (*n*)	10	5	5	1
Drinking history (*n*)	9	5	4	1
Time from ischemic onset to admission (*n*)				
1 day	32	14	18	
2 days	4	4	0	0.23
3 days	6	3	3	

**TABLE 2:** Comparison of the changes of the NIHSS and mRS scores between two groups.

Variable	Without RIPostC (*n* = 21)	With RIPostC (*n* = 21)	*p*
Admission NIHSS score	18 [10,24]	19 [12,21.5]	0.59
3‐day after‐treatment NIHSS score	22 [9,28]	20 [13.5,25.5]	0.94
7‐day after‐treatment NIHSS score	24 [9.5,30]	17 [11.5,23.5]	0.38
90‐day after‐treatment mRS score	5 [3.5,6]	3 [1.5,5.5]	**0.03**

*Note*: Bold indicates stasticallly significant value.

Abbreviations: mRS, modified Rankin Scale; NIHSS, National Institutes of Health Stroke Scale.

**FIGURE 1:**
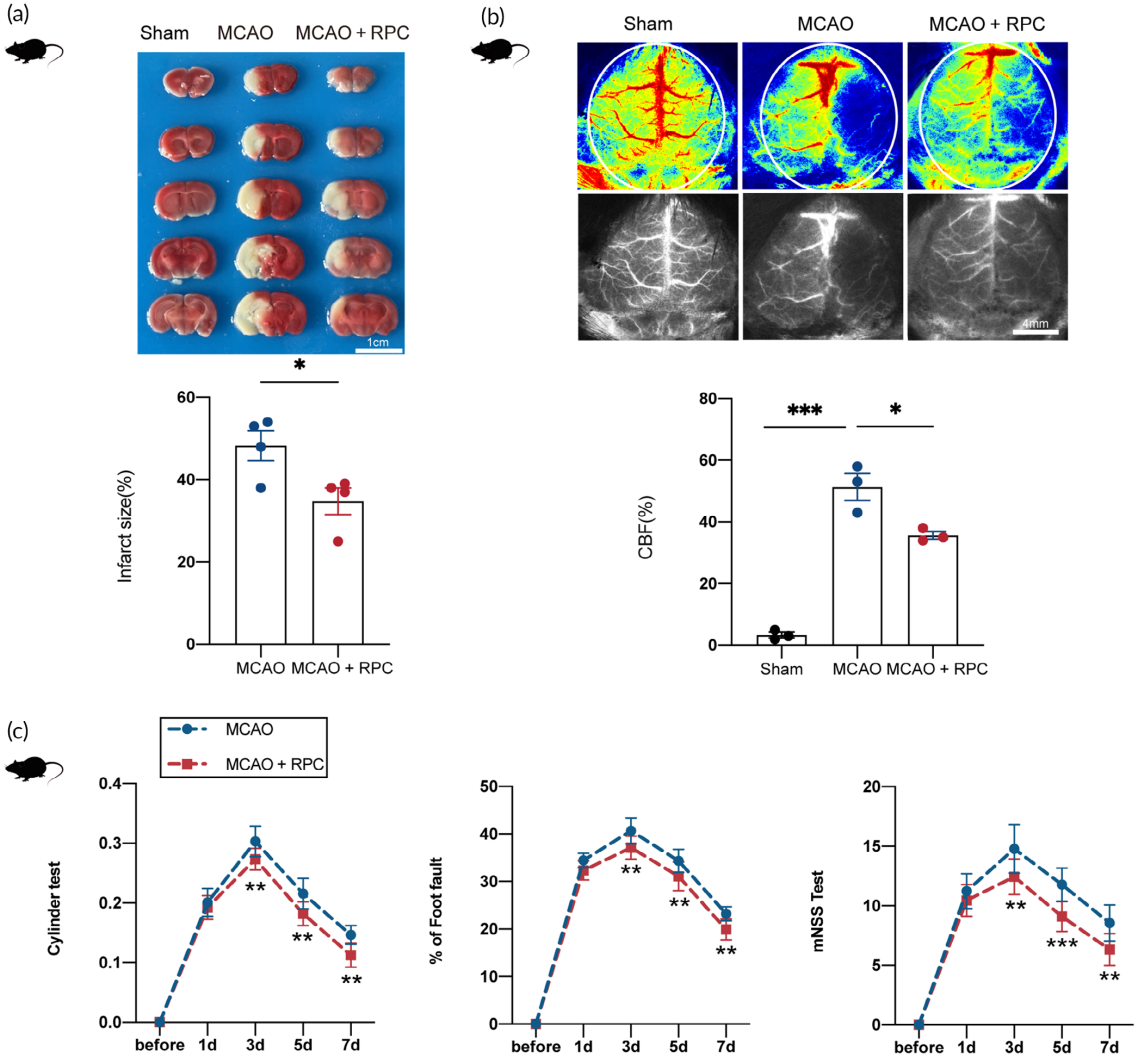
RIPostC reduced infarct size and ameliorated neurological behavior after MCAO. (a) TTC‐stained coronal sections at 3 days after reperfusion from representative animals and infarct volumes and reproducible infarct volumes were observed (Student's *t*‐test, *n* = 4). (b) Representative laser speckle images showing cerebral blood flow at 72 h after reperfusion (One‐way ANOVA, *n* = 3). (c) The foot‐fault test, cylinder test, and mNSS test at different after reperfusion (two‐way ANOVA, *n* = 9). RPC is the abbreviation of RIPostC in the figure. Data presented as mean ± SEM. *p < 0.05; **p < 0.01; ***p < 0.001.

A middle cerebral artery occlusion (MCAO) model was used to reproduce cerebral ischemia injury. Once‐daily bilateral RIPostC was started immediately after MCAO, and it both reduced the infarct size (Figure 1a) and improved cerebral blood flow (Figure 1b) compared with MCAO group mice at 3 days after reperfusion. Next, the effects of RIPostC on neurological improvement were assessed as illustrated in Figure 1c. Compared with the MCAO group, neurological function showed significant improvements after RIPostC.

### 2.2 RIPostC inhibited NLRP3 inflammasome activation and pyroptosis of microglia after AIS

**FIGURE 2:**
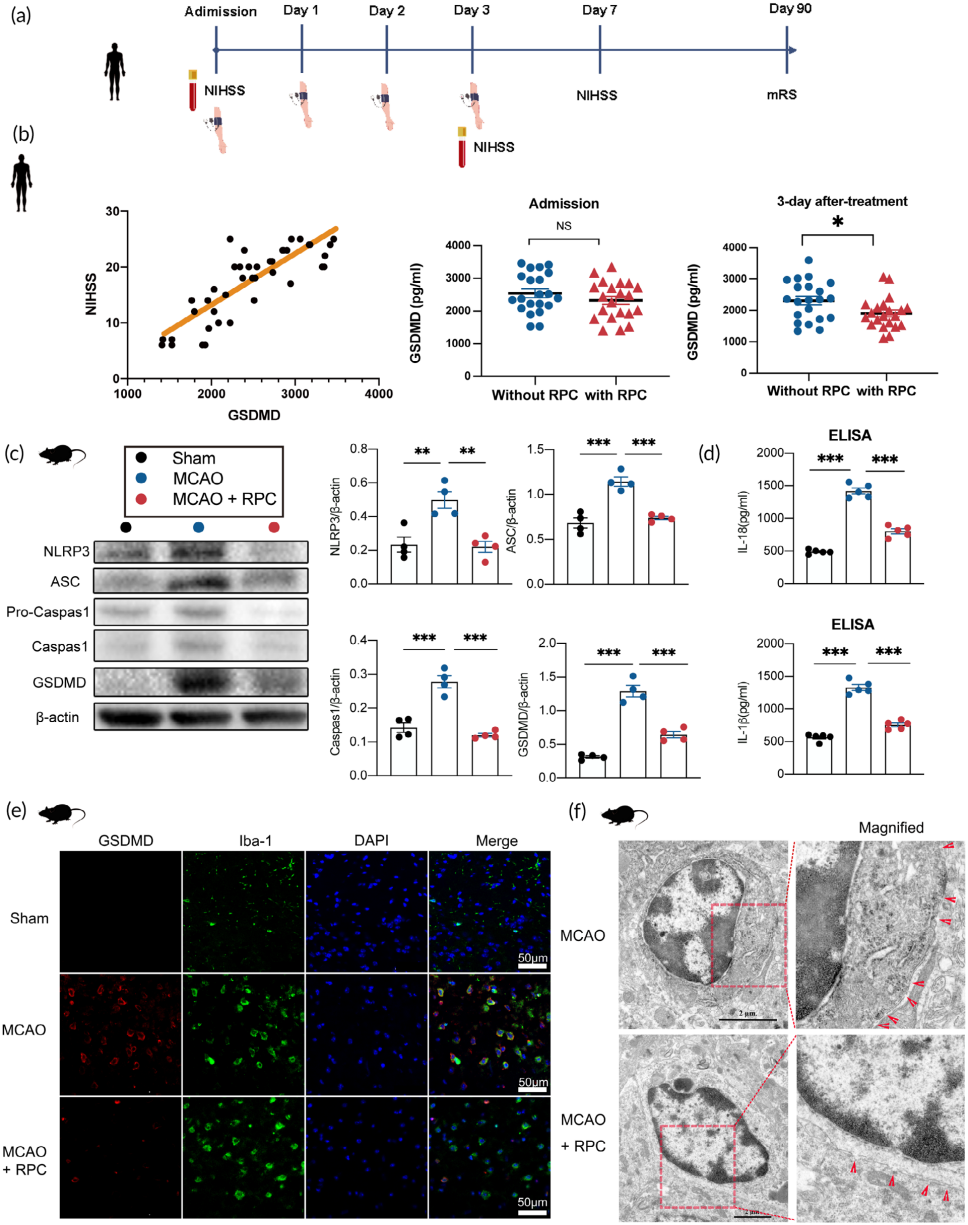
RIPostC triggered NLRP3 inflammasome activation and pyroptosis of microglia 3 days after AIS. (a) Illustration of the clinical experimental timeline. (b) The Pearson linear correlation analysis of the relevance between GSDMD protein expression and NIHSS score (*r* = 0.83, *p* < 0.001). Relative protein expression of GSDMD was assessed by ELISA in 21 AIS patients with RIPostC treatment and 21 AIS patients without RIPostC (Student's *t*‐test, *n* = 21). (c) Representative Western blot images of NLRP3, ASC, Pro‐Caspase 1, Caspase‐1, and GSDMD‐N in ischemic penumbra, as well as quantitative analysis of these proteins in determined with β‐actin for normalization (one‐way ANOVA, *n* = 4). (d) ELISA assays for IL‐1β and IL‐18 in the ischemic penumbra zone of brain tissues (*n* = 5). (e) Double immunostaining of Iba‐1 and GSDMD revealed a good co‐localization of these two makers. Treatment with RIPostC reduced GSDMD positive microglia in ischemic penumbra. bar = 50 μm (one‐way ANOVA, *n* = 3). Data presented as mean ± SEM. (f) Representative transmission electron microscopy images of microglia in peri‐infarct area. Magnified views of microglial cytomembrane are marked with dashed line boxes. Bar = 2 μm. Red arrowhead: membrane pores. RPC is the abbreviation of RIPostC in the figure. *p < 0.05; **p < 0.01; ***p < 0.001.

A total of 42 AIS patients' serums at admission and 3 days after treatment were subjected to ELISA assay, linear correlation analysis confirmed a positive correlation between GSDMD expression and NIHSS score at admission (Figure 2b), indicating the expression level of GSDMD in AIS patients correlated with the severity of the injury. Moreover, quantitative analysis showed no significant difference in GSDMD expression between patients in the two groups at admission (Figure 2b). In contrast, 3 days after treatment, the GSDMD levels were significantly lower in with RIPostC group than in the without RIPostC group (Figure 2b).

Next, we investigated whether microglia pyroptosis is affected by RIPostC after MCAO. Western blot results showed that when compared with the Sham group, MCAO increased the expression of proteins, including NLRP3, ASC, caspase‐1, and GSDMD, which were drastically decreased after RIPostC therapy (Figure 2c). ELISA assay demonstrated that the extracellular expressions of IL‐1β and IL‐18 were increased after MCAO while RIPostC application dampened the release of IL‐1β and IL‐18 (Figure 2d). Double immunofluorescence results implied that an increased amount of GSDMD‐positive microglial cells were detected in the peri‐infarct region of MCAO mice compared with that in Sham mice, which were remarkably attenuated by RIPostC treatment (Figures 2e and S1). As pyroptosis is characterized by the formation of membrane pores (small openings or disruptions in the cell membrane),^25^, ^26^, ^27^ a transmission electron microscope was employed to detect pores formed by GSDMD on the microglia membrane. As displayed in Figure 2f, compared with MCAO mice, the frequency of the membrane pores was reduced after RIPostC.

### 2.3 RIPostC upregulated the expression of HGF after AIS

**FIGURE 3:**
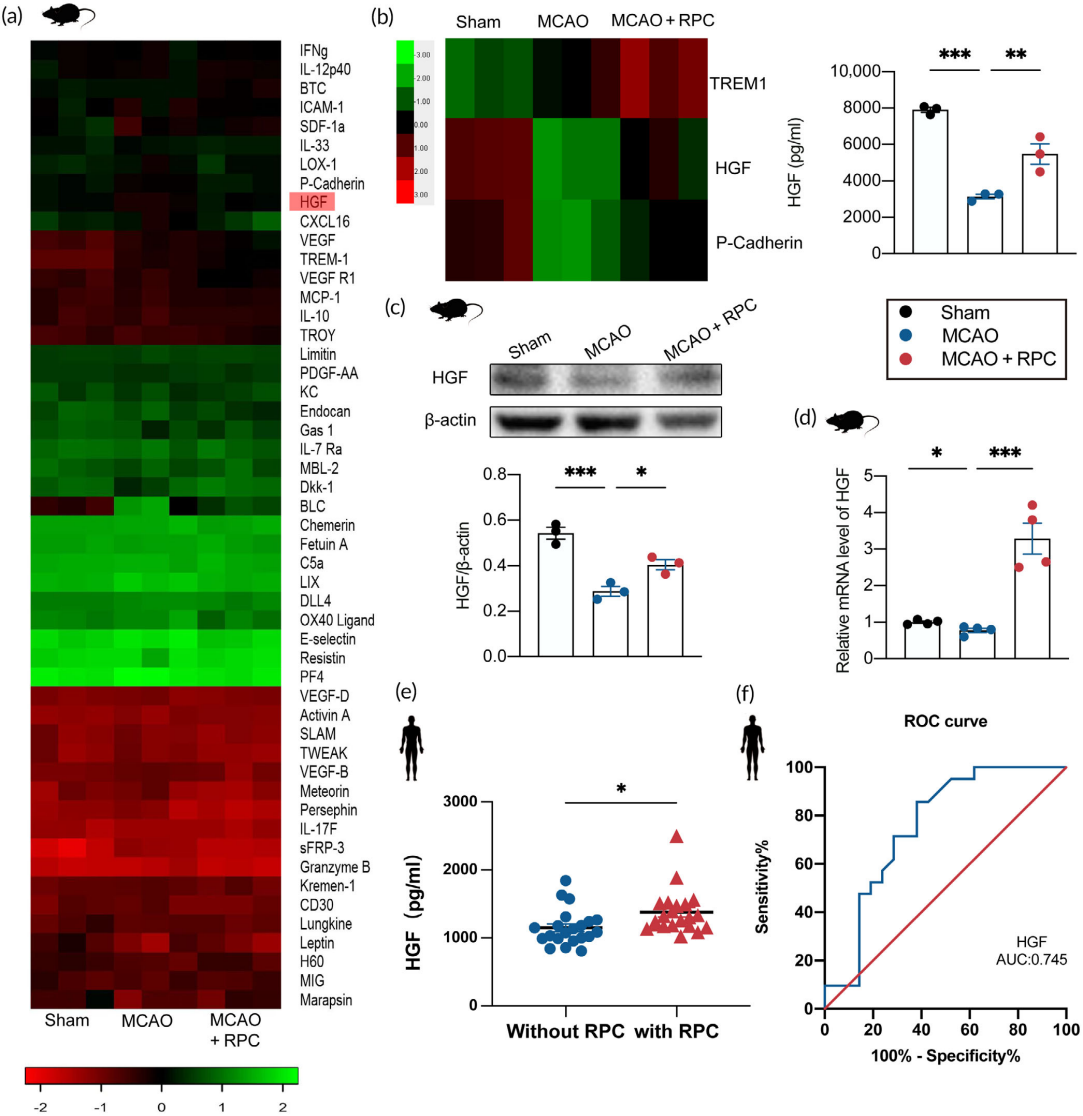
RIPostC increased the level of HGF after AIS. (a) Heat map of protein expression profiles in mice serum with *p* values <0.05 from sham, MCAO and MCAO + RIPostC group (one‐way ANOVA, *n* = 3). (b) Heat map of three cytokines (TREM1, HGF, P‐Cadherin) in mice serum. The analysis of HGF expression in protein chips. (c) The level of HGF in brain tissue of mice was detected by Western blot (*n* = 3). (d) The level of HGF in brain tissue of mice was detected by Real‐time PCR (one‐way ANOVA, *n* = 4). (e) HGF protein expression was assessed by ELISA in AIS patients with or without RIPostC (Student's *t*‐test, *n* = 21). (f) The ROC curve analysis of the diagnostic significance of HGF (AUC = 0.745) for RIPostC treatment (*n* = 21). RPC is the abbreviation of RIPostC in the figure. Data presented as mean ± SEM. *p < 0.05; **p < 0.01; ***p < 0.001.

Then, we investigated the mechanisms by which core functional factors played important roles in RIPostC‐modulated effects on microglia pyroptosis. The high throughput screening protein chip was used to detect the quantity of expression of 200 known cytokines in the serum of mice from three different groups (Sham, MCAO, and MCAO + RIPostC). A clustered heatmap was depicted as a useful visual tool to intuitively examine the expression profile of the 200 cytokines analyzed. From the general microarray, the heat map included only cytokines with *p* values <0.05 (Figure 3a). In Figure 3, intensity values of detection for each cytokine are indicated in color‐coded blocks, with red being positive values, black being zero, and green being negative values, with brighter intensities to express higher levels. Negative values indicated downregulation, and positive values indicated upregulation. Examining the results obtained from microarray analysis, three cytokines (TREM1, HGF, and P‐cadherin) were selected for further analysis (Figure 3b) according to the following criteria: (1) logFC > log2(1.2), at least a 1.2‐fold difference in expression between MCAO and MCAO + RIPostC versus Sham group; (2) the corrected *p*‐value <0.05; (3) the average (fluorescence) signal value of each group was selected at >150 as recommended. Among the three proteins, HGF displayed the most obvious changes (Figures 3b and S2). Real‐time PCR and Western blot were used to verify the brain tissue samples of the mice in the above groups. The results revealed that HGF levels in brain tissues of mice treated with RIPostC were significantly higher than that of MCAO group, consistent with the results of the protein chip (Figure 3c,d). To detect the HGF levels in AIS patients' serums, ELISA was performed. The results revealed that RIPostC increased the protein expressions of HGF in AIS patients (Figure 3e). Also, the ROC curve revealed its potential as a pharmacodynamics blood biomarker for RIPostC treatment (Figure 3f). Therefore, given the above results, we speculated that HGF might participate in RIPostC‐suppressed pyroptosis of microglia after AIS.

### 2.4 HGF/ISG15 is responsible for RIPostC‐attenuated NLRP3‐dependent microglia pyroptosis after AIS

HGF increases the expression levels of ISG15.^23^ After ISG15 removal in human microglia, the expression levels of NLRP3 and IL‐1β were significantly upregulated.^24^ To evaluate the role of HGF/ISG15 in RIPostC‐attenuated NLRP3‐dependent microglia pyroptosis after AIS, we performed HGF and ISG15 silencing using siRNA. Western blot results (Figure S3) showed that the i.c.v. injection of HGF siRNA and ISG15 siRNA significantly reduced the expressions of HGF and ISG15, respectively, both in MCAO and MCAO + RIPostC mice brains, thus demonstrating the knockout efficacy of HGF siRNA and ISG15 siRNA.

**FIGURE 4:**
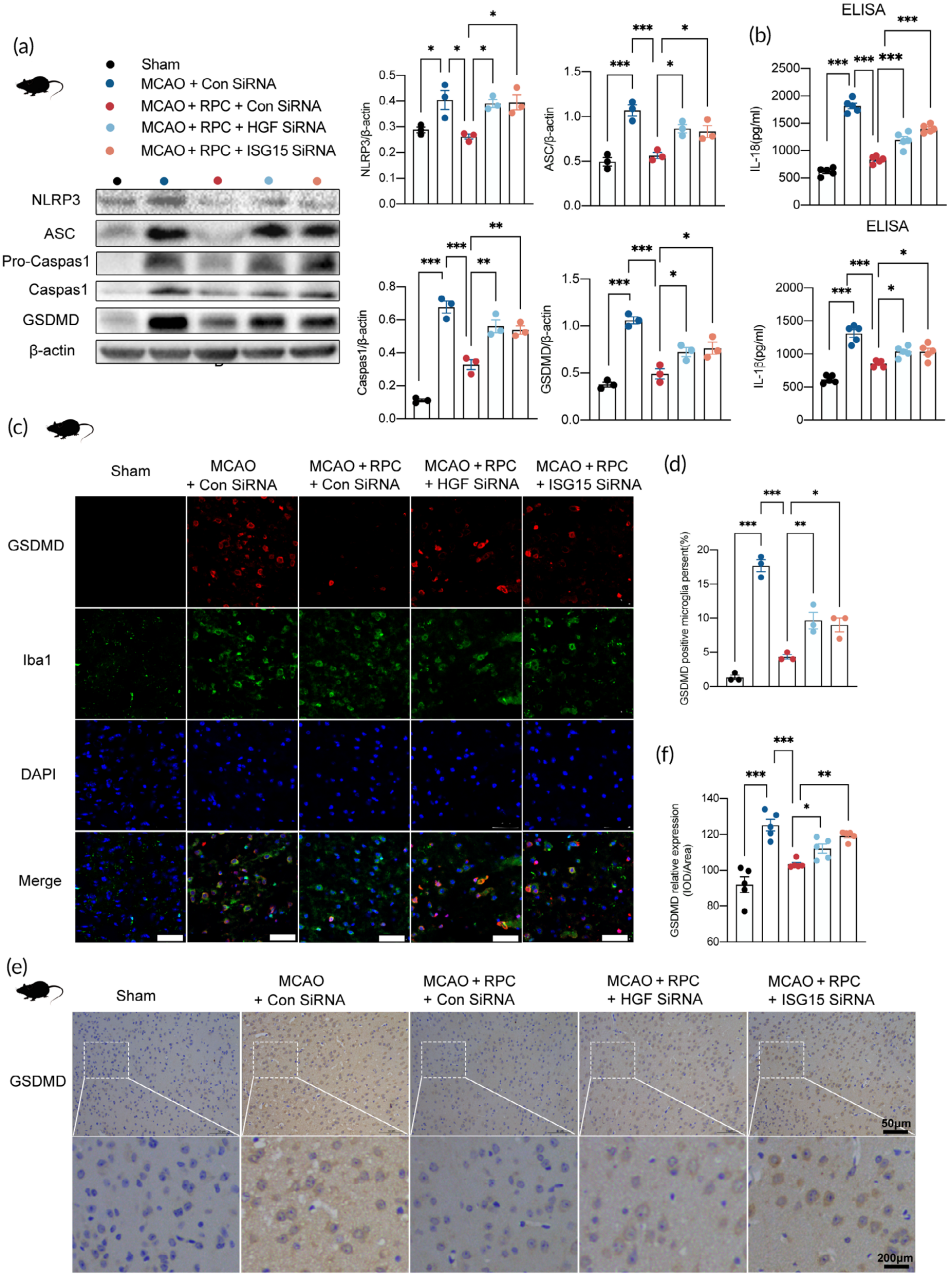
HGF/ISG15 is necessary for RIPostC‐attenuated microglia pyroptosis after MCAO. (a) NLRP3, ASC, caspase‐1, GSDMD protein expressions in ischemic penumbra tissues were detected by western blotting 3 days in RIPostC mice with or without HGF siRNA and ISG15 siRNA treatment (one‐way ANOVA, *n* = 3). (b) The release of IL‐1β and IL‐18 in different treated groups measured by ELISA (*n* = 5). (c,d) Double immunofluorescence showed that both HGF siRNA and ISG15 siRNA treatment notably reversed the decline of GSDMD‐positive microglial cells induced by RIPostC. Bar = 50 μm (one‐way ANOVA, *n* = 3). (e,f) Immunopositivity particles for GSDMD were performed in different treated groups. Bar = 50 μm (one‐way ANOVA, *n* = 5). RPC is the abbreviation of RIPostC in the figure. Data presented as mean ± SEM. *p < 0.05; **p < 0.01; ***p < 0.001.

Next, Western blot results revealed that either HGF siRNA or ISG15 siRNA administration significantly blocked the decremental effects of RIPostC on the expression of proteins, including NLRP3, ASC, caspase‐1, and GSDMD after MCAO (Figure 4a). ELISA assay demonstrated a significant enhancement in the extracellular expressions of IL‐1β and IL‐18 in HGF siRNA or ISG15 siRNA administration compared with MCAO + RIPostC + Con siRNA group (Figure 4b). Additionally, double immunofluorescence results displayed that both HGF siRNA and ISG15 siRNA treatment notably reversed the decline of GSDMD‐positive microglial cells induced by RIPostC (Figure 4c,d). Importantly, immunohistochemistry results consistently proved that when compared with MCAO + RIPostC + Con siRNA group, GSDMD expression was upregulated after applying HGF siRNA or ISG15 siRNA (Figure 4e,f). Taken together, these results displayed that HGF/ISG15 is necessary for RIPostC‐attenuated microglia pyroptosis after MCAO.

### 2.5 HGF/ISG15 attenuated microglia pyroptosis after OGD/R in vitro

**FIGURE 5:**
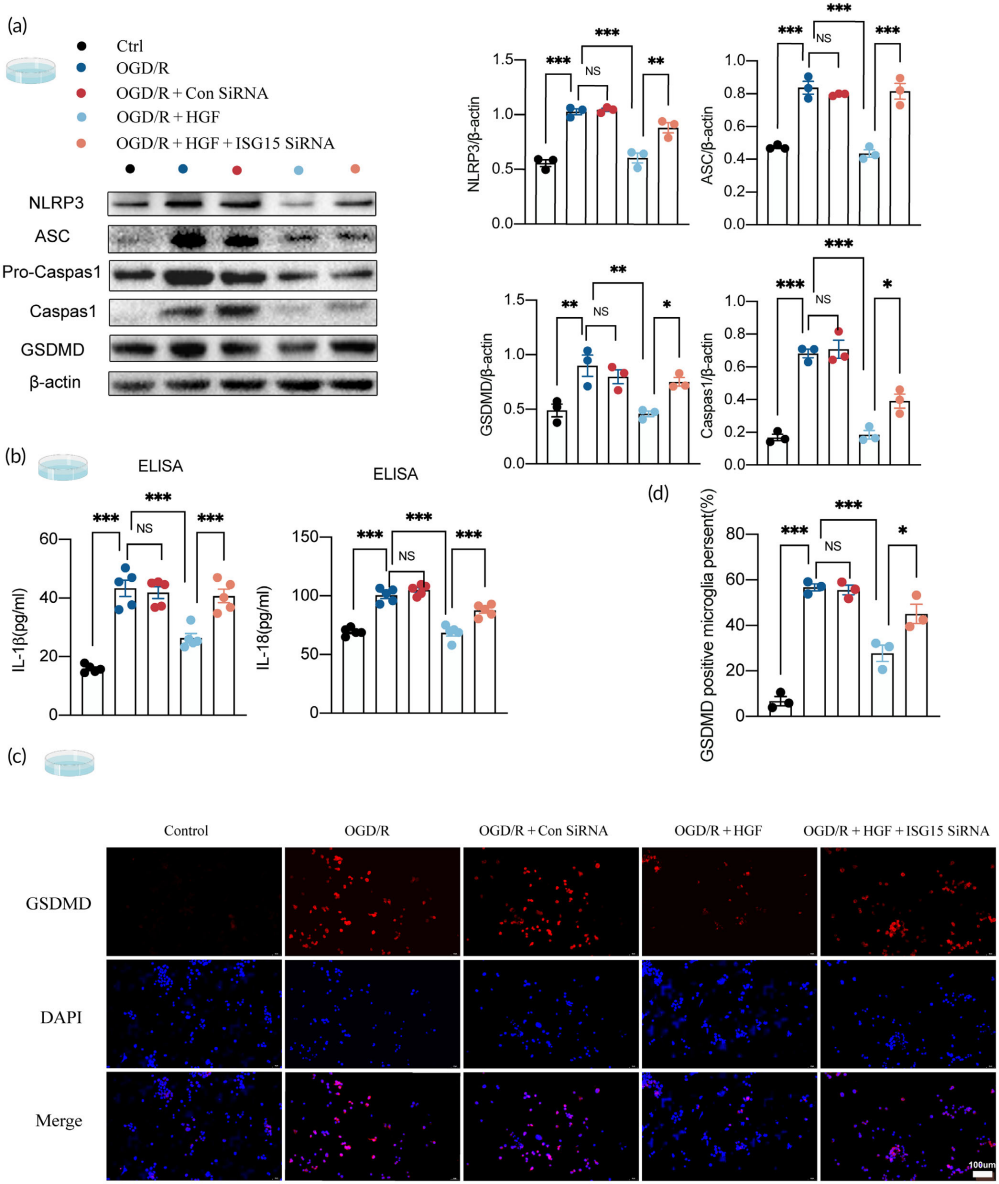
HGF attenuates microglia pyroptosis through ISG15 in vitro. (a) The expression levels of NLRP3, ASC, Pro‐Caspase 1, Caspase‐1, and GSDMD in microglial cells were detected by Western blotting in different treated groups (one‐way ANOVA, *n* = 3). (b) The release of IL‐1β and IL‐18 in different cultured groups measured by ELISA (one‐way ANOVA, *n* = 5). (c,d) Representative images of immunofluorescence of GSDMD acquired from different treated microglial cells (one‐way ANOVA, *n* = 3). All data are presented as mean ± SEM. *p < 0.05; **p < 0.01; ***p < 0.001.

To enhance cell specificity, primary microglia was chosen as the in vitro research model. The oxygen–glucose deprivation/reoxygenation (OGD/R) model was utilized to simulate ischemia–reperfusion injury in the brain, thereby inducing microglia pyroptosis. Additionally, Exogenous HGF was applied to simulate the upregulation of HGF by RIPostC. A total of 40 ng/mL HGF was used to effectively increase the expression of HGF for the following experiments (Figure S4). To observe the influence of ISG15 inhibition on HGF under OGD/R conditions, ISG15 siRNA was performed based on HGF treatment. Figure S5 demonstrates the knockout efficacy of ISG15 siRNA. We then evaluated whether HGF/ISG15 influenced microglia pyroptosis under the OGD/R model. Our results revealed that the expression of NLRP3, ASC, caspase‐1, and GSDMD was enhanced in OGD/R microglia, which was abolished by HGF treatment, whereas these reductions were abrogated by applying ISG15 siRNA (Figure 5a). ELISA assay demonstrated that HGF treatment markedly downregulated OGD/R‐induced elevation of extracellular IL‐1β and IL‐18, which were reversed by ISG15 siRNA (Figure 5b). Immunofluorescence results suggested that after HGF therapy, fewer GSDMD‐positive microglial cells were detected compared with OGD/R group, which were remarkably prevented by ISG15 siRNA treatment (Figure 5c,d). Therefore, consistent with the observation in vivo, HGF/ISG15 showed the ability to alleviate NLRP3 inflammasome activation and pyroptosis of microglia in vitro.

## 3 DISCUSSION

In the present article, we identified for the first time a protein scripture of ischemic blood subjected to the protective strategy termed remote limb ischemic postconditioning (RIPostC). In particular, we were able to demonstrate the following: (1) GSDMD protein in the blood samples from AIS patients was correlated with the severity of this disease while RIPostC treatment could alleviate the level of GSDMD of AIS patients. (2) The upregulation of HGF in AIS patients and mice subjected to RIPostC. (3) RIPostC could inhibit the expression of NLRP3, ASC, caspase‐1, GSDMD, IL‐1β, and IL‐18, leading to decreased microglial pyroptosis in AIS mice. (4) Either HGF siRNA or ISG15 siRNA intracerebroventricular administration notably blocked the inhibition effects of RIPostC on microglia pyroptosis in AIS mice. (5) Exogenous HGF attenuated microglia pyroptosis in AIS cell model, whereas these reductions were abrogated by ISG15 siRNA. Collectively, RIPostC inhibits NLRP3‐mediated pyroptosis after AIS, at least partly through the HGF/ISG15 pathways.

Ren et al. were the first researchers to discover that RIPostC performed on the ipsilateral hind limb reduced the cerebral infarct size after focal cerebral ischemia.^28^ Until now, RIPostC is a well‐established neuroprotective strategy for preventing ischemic stroke‐induced brain injury.^29^, ^30^, ^31^ Consistent with prior studies, our findings indicated that RIPostC decreased cerebral infarct size, improved cerebral blood flow, and promoted neurological role at day 3 post‐stroke (Figure 1). While RIPostC is being tested in clinical ischemic stroke studies worldwide, the mechanisms activated by RIPostC as involved in neuroprotection are yet to be completely elucidated.^30^, ^32^

Brain damage following AIS injury is a complicated pathophysiological issue in which inflammatory responses have been suggested to play an important role.^33^ Pyroptosis is gasdermin‐mediated programmed necrosis characterized by proinflammatory activity leading to cell lysis and inflammation.^34^, ^35^ This study included 42 AIS patients, comprising 21 patients with RIPostC and 21 patients without RIPostC. Throughout the study period, all patients received foundational treatment. RIPostC was performed twice daily from the time of enrollment to day 3 and serums were collected to detect the GSDMD levels. The comparison of NIHSS scores between patients in the two groups revealed no statistically significant difference at 3 days and 7 days after treatment. However, patients in the RIPostC group had significantly lower mRS scores, as compared with the without RIPostC group at 90 months after treatment (Table 2) Thus, RIPostC group patients reported significantly improved scores in functional outcome evaluation than the without RIPostC group patients. Moreover, linear correlation analysis showed that GSDMD protein level was correlated with the severity of AIS (Figure 2b). RIPostC treatment was noted to reduce GSDMD protein levels in AIS patients (Figure 2b).

Several studies have found that pyroptosis mainly occurs in the microglia in CNS neuroimmune diseases,^36^, ^37^, ^38^ and microglia pyroptosis serves as a key executioner in cerebral ischemia.^14^, ^39^ However, knowledge about microglia pyroptosis in RIPostC after AIS has not been reported yet. In this study, we demonstrated the first evidence that RIPostC application suppressed the GSDMD immunostaining in Iba‐1‐labeled microglia and protein expression of GSDMD‐N in MCAO mice (Figure 2c,e). Studies showed that the N‐terminal fragment of GSDMD could bind to phosphoinositide of the plasma membrane and form a 12–14 nm membrane pore, which is the characteristic structure of pyroptosis.^17^ Indeed, we found that RIPostC decreased GSDMD pores in the microglia membrane (Figure 2f), thus these data indicate that a pyroptosis occurring in microglia could be inhibited by RIPostC. The NLRP3 inflammasome has been reported to be the main member expressed in microglia and a key mediator that plays an important role in pyroptosis.^40^, ^41^ Upon activation, the NLRP3 inflammasome results in caspase‐1 activation, which induces maturation of the cytosolic inflammatory cytokines, IL‐1β, and IL‐18. It also causes cleavage of cytosolic GSDMD to mediate pyroptotic cell lysis, thus leading to spillage of intracellular cytokines and alarmins.^14^, ^42^ These cytokines are crucial in triggering and amplifying inflammatory responses, which can cause cell death or brain injury.^15^ In our previous study done on mice, we found that NLRP3 blockade inhibited neuronal apoptosis and improved neurological outcomes after ischemic stroke.^43^ Here, Western blot and ELISA results displayed that the protein expressions of NLRP3, ASC, cleaved caspase‐1, IL‐1β, and IL‐18 were reduced by RIPostC after MCAO (Figure 2c,d). Viewed together, these results indicated that NLRP3‐dependent microglia pyroptosis could be inhibited by RIPostC in AIS. A previous study^9^ has reported that RIPC can inhibit the inflammatory reaction by promoting microglia/macrophage transferring from M1 to M2 phenotype after MCAO/R in mice. Consistent with the findings of this study, our investigation verified the inhibitory effect of RIPC on post‐stroke inflammatory response from the perspective of microglial pyroptosis, a specific form of inflammatory cell death. In this study, the reason we focus on the effect of RIPostC on microglia pyroptosis rather than other types of cells is that microglia are vital mediators of innate immune responses following AIS and these cells are also considered to be the main cells in CNS where pyroptosis occurs.^36^, ^37^, ^44^ However, we do not deny that other types of cells also exist in pyroptosis and their role in the progression of AIS is still worthy of further study.

RIPostC is a powerful endogenous protective mechanism that, when applied to the body, promotes the production of numerous endogenous protective substances to counteract ischemia–reperfusion injury.^45^ Next, we examined which core functional factors induced by RIPostC played important roles in the effects of RIPostC on microglia pyroptosis after AIS. High throughput screening (HTS) was used to detect the quantity of expression of 200 known cytokines in the serum of mice. Based on the above‐mentioned standards, three cytokines were selected, namely TREM1, HGF, and P‐Cadherin (Figure 3a,b). Among the three factors, HGF demonstrated the most significant changes. Real‐time PCR and Western blot revealed that the expression levels of HGF in the brain tissue of MCAO mice treated with RIPostC were significantly higher than that of MCAO group, consistent with protein chip results (Figure 3c,d). To verify the clinical application of our experimental results, we detect the level of HGF in serums of 42 patients, ELISA showed that HGF was significantly upregulated after RIPostC treatment (Figure 3e). ROC curve showed that HGF may be a biomarker for the RIPostC treatment in AIS patients (Figure 3f). Therefore, HGF is the most prominent endogenous protective factor produced by the body in response to ischemia–reperfusion injury after RPC application. Although the exact mechanism by which HGF is transferred from the remote site to the brain remains unclear, preclinical studies suggest that the mechanisms of RIC involve a combination of circulating humoral factors and neuronal signals.^45^ It could also potentially become a direction for future research. Considering the results of our study mentioned above, we focused on HGF in subsequent studies. HGF has a protective function as it reduces neuroinflammation in AIS.^20^ Interestingly, HGF has been reported could inhibit septic endothelial pyroptosis,^22^ therefore, we speculate whether HGF plays a key role in the effect of RIPostC on microglia pyroptosis after AIS. Our results indicated that HGF siRNA administration blocked RIPostC‐mediated repression on NLRP3 inflammasome activation and microglia pyroptosis in MCAO mice (Figure 4). As a pharmacological mimic for RIPostC‐mediated HGF upregulation, exogenous HGF was applied in OGD/R microglia. It was observed that HGF treatment prevented NLRP3 inflammasome activation and pyroptosis following OGD/R (Figure 5). HGF has been reported to increase the expression level of ISG15 in endothelial cells,^23^ and ISG15 has been found to inhibit the release of IL‐1β in microglia.^24^ Consistent with these studies, we found that ISG15 siRNA administration reversed the inhibition effect of RIPostC (Figure 4) or exogenous HGF on microglia pyroptosis (Figure 5). Given this, HGF/ISG15 is necessary for RIPostC‐attenuated microglia pyroptosis in ischemic injury.

**FIGURE 6:**
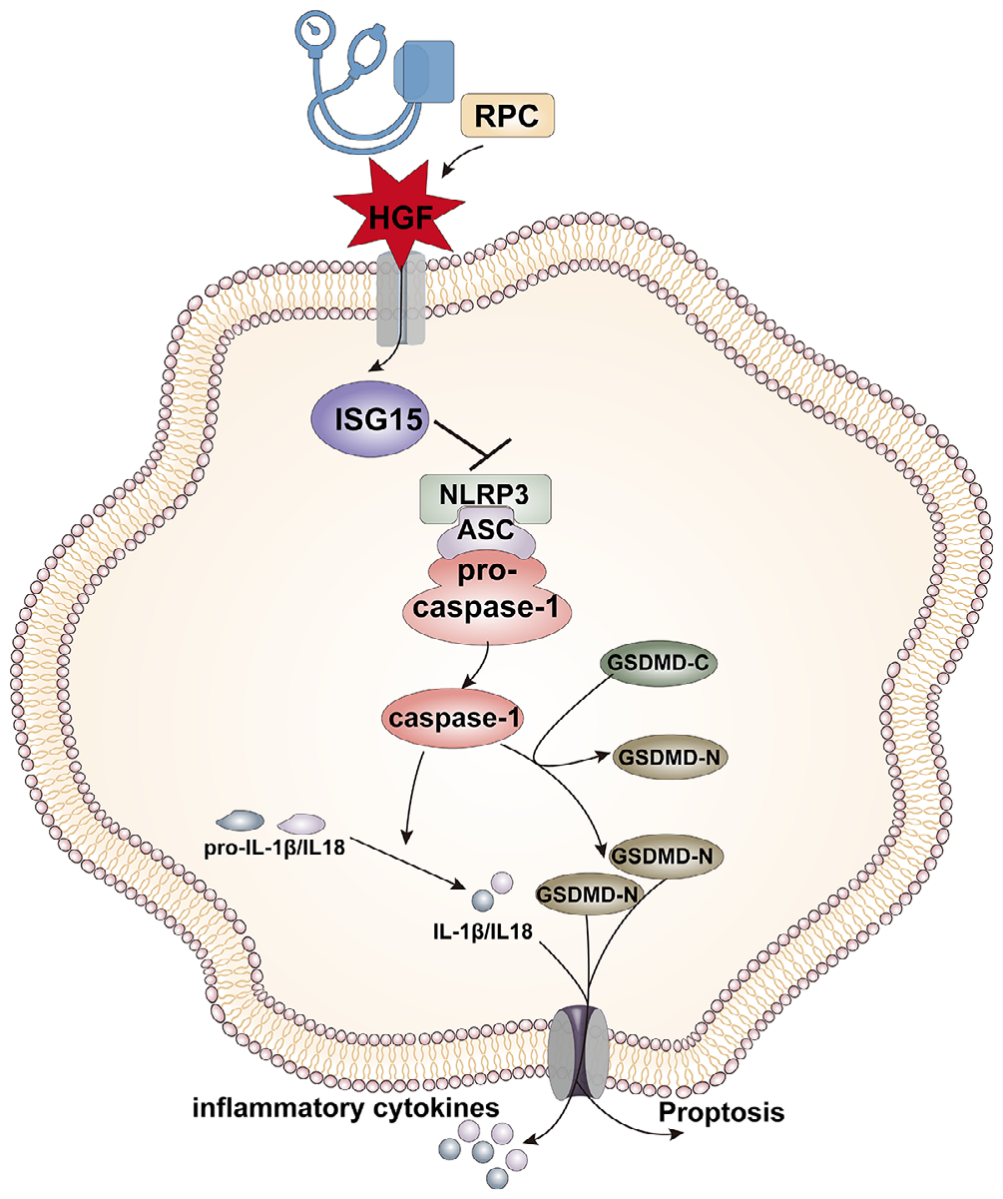
RIPostC overcame NLRP3‐Dependent microglia pyroptosis may involve the HGF. We demonstrated that RIPostC, a noninvasive protection strategy, can inhibit microglia pyroptosis via mediating GSDMD through HGF/ISG15 axis.

In summary, the present study demonstrates for the first time that RIPostC plays an antipyroptosis role in AIS, which at least partially involves the HGF/ISG15 axis (Figure 6). Not only does this finding suggest the pharmacological efficacy of RIPostC, but also it provides evidence for the clinical application of RIPostC in AIS. Moreover, we propose that HGF has the potential to be utilized as a target biomarker in RIPostC treatment.

## 4 MATERIALS AND METHODS

### 4.1 Donor recruitment and blood sample preparation

In this study, a randomized single‐blind approach was employed to enroll 42 AIS patients from the Affiliated Hospital of Xuzhou Medical University between November 2021 and June 2022. The inclusion criteria included the following: (1) patients aged 18–85 years old; (2) NIHSS score of ≤25 and NIHSS >5; (3) RIPostC was started within 3 days after ischemic onset; (4) written informed consent signed by patients or their families. Patients who had received thrombolysis or endovascular therapy with other intracranial lesions, such as cerebral hemorrhage, cerebral venous disease, severe subclavian artery stenosis, and other brain diseases, were excluded. The National Institutes of Health Stroke Scale (NIHSS) and modified Rankin Scale (mRS) were used to assess the severity of AIS. Total scores on the NIHSS ranged from 0 to 42, and higher scores reflect more severe neurological disability. The poststroke functional limitations were the second efficacy outcome measure of the trial, which was evaluated by the mRS on day 90. mRS is a validated measure of functional outcome after stroke with potential scores from 0 (indicating no functional limitation) to 5 (indicating severe functional disability); a score of 6 denotes death.

After informed consent has been given, the simple randomization method was performed, where participants have an equal chance of being assigned to either the RIPostC or control group. Throughout the study period, all patients in the control and RIPostC groups received foundational treatment, including blood vessel expansion, free radical elimination in the acute stage, blood pressure and blood glucose stabilization, as well as antiplatelet and lipid‐lowering drugs. All treatments strictly adhered to guideline recommendations and were adjusted according to each patient's situation. RIPostC was performed twice daily from the time of enrollment to day 3. An automatic device (LiShangSong Medical Devices, China) was used in the upper arm of the less paralyzed side. The whole intervention process consisted of four cycles of extremity ischemia involving 5 min of blood pressure cuff inflation to 200 mmHg, followed by 5 min cuff deflation.

For all participants, serum samples were isolated from venous blood at admission and 3 days after treatment to measure the levels of HGF and GSDMD, using the enzyme‐linked immunosorbent assay (ELISA) kit (Meimian, China) according to the manufacturer's instructions. Data collection and outcome assessment were performed only by members blinded to group allocation.

### 4.2 Animal mortality and exclusion

The overall mortality was 9.47% (20/211). None of the sham‐operated mice died in this study. The mortality did not significantly differ among the experimental MCAO groups. None of the MCAO mice were excluded (Table S1). No significant adverse effects were observed in treatment groups.

### 4.3 Mice

Biological sex plays a crucial role in determining the outcome of strokes. Female animals have relative protection against cerebrovascular diseases compared with males.^46^ To minimize the influence of sex, male C57BL/6 mice aged 2–3 months (20–25 g) were used in all animal experiments. All experiments in this study were approved by the Institutional Animal Use and Care Committee of Xuzhou Medical University. The mice were housed in individual cages with a 12 h light/dark cycle and had free access to food and water.

### 4.4 Induction of the MCAO model in mice

Mice cerebral ischemia/reperfusion (I/R) models were induced by MCAO surgery using the intraluminal filament. Under isoflurane anesthesia, the right external carotid artery (ECA) was ligated, and a silicon‐coated monofilament nylon suture (diameter: 0.20 ± 0.01 mm, RWD Life Science, China) was inserted into the ECA and advanced into the internal carotid artery (ICA) until mild resistance was felt. The filament was removed after 1 h to initiate reperfusion. During the surgery, core body temperatures were continuously maintained at 36.8°C–37.2°C. For the control group, the same surgery was performed except for MCA occlusion.

### 4.5 Remote limb ischemic postconditioning

Mice RIPostC was performed as previously described using a simple randomization method.^9^ Bilateral hind limb ischemia (10 min–10 min/cycle) was conducted immediately after MCAO and continued daily after that, under anesthesia with 1%–3% isoflurane. During every cycle, the proximal regions of the hind limbs were secured using a tourniquet for 10 min, followed by reperfusion for 10 min with the tourniquet released. MCAO groups contained all surgical techniques (including anesthesia with the same dosage of isoflurane), excluding bilateral hind limb ischemia and reperfusion cycles. The experimenters were responsible for administering the treatments were blinded to the RIPostC treatments.

### 4.6 Measurement of infarct size

The mice were euthanized, and their brains were promptly extracted 72 h after surgery. Each brain was dissected and sectioned into five coronal slices at 2 mm intervals. The brain slices were placed in 1% 2,3,5‐triphenyltetrazolium chloride (TTC, Sigma, Germany) solution and incubated for 10 min in the dark at 37°C. ImageJ software was used to evaluate the infarct size on both sides of each section. According to this equation, the infarct size was represented as an average percentage from five slices: infarct size (%) = (contralateral area − ipsilateral non‐infarct area)/contralateral area × 100%.

### 4.7 Laser speckle contrast imaging

In brief, the mice were anesthetized, and a midline incision was performed. Perfusion images were acquired using PeriCam high‐resolution laser speckle contrast imaging (LSCI), and the acquired images were analyzed for dynamic changes in cerebral blood flow. Regional cerebral blood flow reduction rate = (ROI1 − ROI2)/ROI1 × 100% (ROI1 represents right cerebral blood flow; ROI2 represents left cerebral blood flow). Body temperature was maintained at 37°C ± 0.5°C throughout the experiment.

### 4.8 Behavior assessment

To evaluate behavior, the mice were tested at 0, 1, 3, 5, and 7 days after operation. All behavioral assessments were conducted by individuals who were blinded to experimental treatments.

### 4.9 The modified Neurological Severity Score

The modified Neurological Severity Score (mNSS) includes motor, sensory, balance, and reflex tests, as well as the corner‐turning test.^47^ The score ranged from 0 to 18 points (normal score of 0; maximal deficit score of 18); mice with more severe damage received higher scores.

### 4.10 Cylinder test

The cylinder rearing test was conducted as described previously.^48^ After placing a mouse individually inside an open‐top transparent hollow acrylic cylinder (10 × 20 × 2 cm), the number of times their forelimb touched against the cylinder wall was counted for 5 min. The use of the right or left forelimb was defined as the placement of the whole palm on the cylinder wall. A total of 20 wall touches with the ipsilateral and the contralateral forelimbs were analyzed. The percentage of ipsilateral or contralateral forelimb usage was used to determine the frequency of forelimb usage.

### 4.11 Foot‐fault test

The mice were placed on a horizontal grid floor elevated above the surface and allowed to walk 100 steps. A foot fault was recorded when the mouse's foot was miss‐stepped on the grid or the foot fell downward through the openings between the grids. All four limbs were observed for misses. The percentage of total foot faults was recorded and analyzed.

### 4.12 Intracerebroventricular injection of siRNA

Before MCAO induction, ISG15 siRNA (Dharmacon, USA), HGF siRNA (Dharmacon, USA), or control scramble siRNA (Dharmacon, USA) were administered using in vivo‐jetPEITM (PolyplusTransfection) via intracerebroventricular injection (positioned 1 mm posterior, 1.0 mm lateral, and 3.2 mm deep relative to the bregma). According to manufacturer's instructions^49^ for in vivo delivery and one mouse, the required amount of nucleic acid and in vivo‐jetPEI was diluted in 5% glucose solution (final concentration) and mixed via a vortex. For the 1 mg/kg condition, 0.12 μL of PEI was used per μg of siRNA. The diluted transfection reagent was added to the siRNA solution, vortex‐mixed, and left for at least 15 min at room temperature. The complexes were injected into mice (4 μL). The control siRNA was non‐targeted scrambled (Polyplus Transfection). To examine the transfection efficiency, a Western blot was performed.

### 4.13 Cell culture and experimental protocols

#### 4.13.1 Primary microglial culture

New‐born C57BL/6 mice brains were removed at days 1–3, cortices were cut into pieces and triturated into single cells. Cell suspensions underwent filtration using a 70‐μm cell strainer. Mixed glial were plated into T‐25 culture flasks containing DMEM/F12 with 10% fetal bovine serum, GlutaMAX (Invitrogen), and 1% penicillin/streptomycin in a 5% CO_2_/37°C incubator. Cell culture medium was changed every 5 days. After 2 weeks, the flasks were shaken at 220 rpm for 4 h at 37°C to harvest the primary microglia. Thereafter, the microglia were plated in 6‐well plates at a density of 5 × 10^5^ cells per well for subsequent experiments. Immunocytochemical staining showed that the primary microglia cells were more than 95% pure.

#### 4.13.2 OGD/R model

For the simulated AIS experiments in vitro, the OGD/R model was utilized. The samples were pretreated with 10, 20, and 40 ng/mL HGF (Peprotech) and subjected to OGD/R treatment before being placed in glucose‐free DMEM and exposed to hypoxia (94% N_2_, 5% CO_2_, and 1% O_2_) at 37°C in a humidified chamber (ThermoFisher Scientific, Inc.) for 3 h, followed by incubation under normal culture conditions for 24 h.

### 4.14 Transfection of siRNA in vitro

The siRNA gene silencing in microglial cells was conducted as previously described.^32^ Briefly, microglial cells were seeded into a 6‐well plate (Corning Inc.) at a density of 1.5 × 10^5^ cells/well 24 h before transfection. The microglial cells were transfected by Lipofectamine 2000 (Invitrogen; Thermo Fisher Scientific, Inc.) with ISG15 siRNA (Dharmacon, USA) or control siRNA (Dharmacon, USA) for 24 h. Western blot was conducted to examine the transfection efficiency.

### 4.15 Quantitative protein chip testing

Blood samples were collected from the venue angularis of mice from different groups (Sham, MCAO, MCAO + RIPostC). The volume of each blood sample was approximately 600 μL. All blood samples were centrifuged, and the supernatants were stored in separate vials at −80°C for batch serum analysis. Differential protein screening was conducted with RayBiotech Mouse Cytokine Antibody Array (RayBiotech, Inc. Norcross, GA; catalog No. QAM‐CAA‐4000), a combination of five non‐overlapping arrays to quantitatively measure 200 mouse cytokines. One standard glass slide was spotted with 16 wells of identical cytokine antibody arrays, and each antibody was arrayed in quadruplicate. Signal intensity values representing detected cytokines were then subtracted from the background and normalized to positive controls on the same membrane. All experimental steps and analyses were conducted according to the manufacturer's instructions.

### 4.16 Quantitative real‐time PCR

Total RNA was extracted from peri‐infarct tissues using TRIzol reagent (Thermo Fisher Scientific, USA) and was reverse transcribed into cDNA with RevertAid First Strand cDNA Synthesis Kit (Thermo Fisher Scientific, USA). Quantitative real‐time PCR was then performed with UltraSYBR Mixture (Vazyme, China), specific mouse primers, and cDNA using Mx3000P Real‐Time PCR System (Agilent Technologies, USA). Expression of the gene of interest was normalized to glyceraldehyde‐3‐phosphate dehydrogenase (GAPDH). The expression levels of genes were measured in terms of threshold cycle value (CT) using the 2^−ΔΔCt^ method.^50^ The primer sequences (Sangon Biotech, China) were as follows: HGF: F‐GCTATCGGGGTAAAGACCTACA, R‐CGTAGCGTACCTCTGGATTGCGAPDH, GAPDH: F‐AGGTCGGTGTGAACGGATTTG, R‐TGTAGACCATGTAGTTGAGGTCA.

### 4.17 Western blot analysis

Protein extraction and Western blot procedures were conducted according to previously described methods.^51^ Total proteins from the ischemic cortical area brain tissues on day 3 after AIS or microglial cells were collected after OGD (3 h)/R (24 h) in equal amounts of cell lysate, and the separated proteins in the supernatant were subsequently transferred. For immunoblotting, the following primary antibodies were used: NLRP3(1:1000; Abcam, UK), ASC (1:1000; Cell Signaling Technology, USA), Caspase‐1 (1:1000; Cell Signaling Technology, USA)，GSDMD (1:500; Santa Cruz Biotechnology, USA), IL‐1β (1:1000; Cell Signaling Technology, USA), IL‐18 (1:1000; Proteintech, USA), HGF (1:800;R&D, USA), and ISG15 (1:500; Santa Cruz Biotechnology, USA).

### 4.18 Immunofluorescence staining

For in vivo studies, the mice were deeply anesthetized on day 3 after surgery and perfused with saline, followed by 4% paraformaldehyde. The brains were gathered and dehydrated in a 30% sucrose solution overnight. Afterward, brains were transferred to OCT and sliced into 10 μm frozen segments. The slices were placed in a blocking buffer containing 90% PBS, 10% donkey serum, and 0.5% Triton X‐100 for 1 h and then treated with a primary antibody: anti‐Iba‐1 (1:500, WAKO, Japan), anti‐GSDMD (1:100; Santa Cruz Biotechnology, USA) overnight at 4°C. Then, these sections were washed three times with PBS, followed by incubation with an Alexa Fluor 592 or Alexa Fluor 488 secondary antibody (1:500, Protein tech Group, USA) at 37°C for 2 h before being mounted with Fluoroshield containing DAPI. ImageJ software was used to quantify the number of target cells.

For in vitro studies, cultured microglia grown on coverslips were fixed with 4% paraformaldehyde in PBS for 30 min, permeabilized for 20 min at room temperature, followed by 15 min incubation with 0.1% Triton X‐100 in PBS, and blocked using 5% BSA for 1 h. Samples were stained with GSDMD (1:100, Santa Cruz Biotechnology, USA) primary antibodies at appropriate dilutions and then incubated with the secondary antibody conjugated with Alexa Fluor 592 (1:500, Protein Tech Group, USA). Finally, the dishes were mounted in Fluoroshield containing DAPI. Image J software was used to quantify the positive signals.

### 4.19 Immunohistochemistry staining

On the third day after the stroke, mice from different groups were perfused with 4% paraformaldehyde. Their brains were removed and fixed in the same fixative overnight. They were then paraffin‐embedded and sectioned into 5 μm coronal slices. This process was followed by deparaffinization with xylene and graded ethanol sequentially. Next, 10 mmol citrate buffer was conducted for antigen retrieval, 3% hydrogen peroxide was applied for endogenous peroxidase activity blockade, and 5% bovine serum albumin was performed for blocking the nonspecific staining. Subsequently, these sections were incubated with anti‐GSDMD (1:100) at 4°C overnight. The sections were then incubated with a secondary antibody for 1.5 h at room temperature, followed by DAB staining for 3 min. Finally, immunopositive cells from three different areas around the hematoma were analyzed by three observers who were blinded to the mice group information. Image J software was used to quantify the positive signals.

### 4.20 Enzyme‐linked immunosorbent assay

Blood samples were obtained from the cubital vein of each participant and centrifuged. Brain tissues from the ischemic penumbra zone were dissected and homogenized. Cell culture supernatants were collected. The concentrations of IL‐1β and IL‐18 proteins were detected using enzyme‐linked immunosorbent assay (ELISA) kits (Jianglai biotech, China) according to the manufacturer's instructions.

### 4.21 Statistical analysis

The data in this study were statistically analyzed using SPSS 26.0 (IBM Corp, USA). We chose animal experimental groups with the smallest sample sizes as representatives for power analysis. With a significance level of 0.05 and a sample size of 3, the power of the analysis was consistently above 0.8, indicating that the conclusions drawn from this study are reliable even with the available sample size (Table S2). All data are presented as the mean ± SEM. One‐way ANOVA with Tukey's post hoc test was applied to conduct comparisons among the multiple groups. The Student's *t*‐test (parametric) or Mann–Whitney *U* test (nonparametric) was used to compare the differences between the two groups. The comparative variation was significant at *p* < 0.05.

## AUTHOR CONTRIBUTIONS

**Lu Yu:** Conceptualization (equal); funding acquisition (supporting); investigation (lead); writing – original draft (lead); writing – review and editing (equal). **Zuohui Zhang:** Methodology (equal); writing – review and editing (supporting). **Hao Chen:** Software (supporting); supervision (supporting); validation (supporting). **Miao Wang:** Data curation (equal); methodology (supporting); supervision (supporting). **Wenqi Mao:** Data curation (supporting); methodology (equal); validation (supporting). **Jinxia Hu:** Funding acquisition (equal); project administration (equal). **Dandan Zuo:** Data curation (supporting); methodology (supporting); software (supporting). **Bingchen Lv:** Conceptualization (supporting); formal analysis (supporting); methodology (supporting). **Weifeng Wu:** Conceptualization (supporting); software (supporting); writing – original draft (supporting). **Suhua Qi:** Conceptualization (supporting); investigation (supporting); project administration (supporting); writing – review and editing (supporting). **Guiyun Cui:** Funding acquisition (equal); project administration (lead); resources (equal); writing – original draft (equal); writing – review and editing (lead).

## FUNDING INFORMATION

The National Natural Science Foundation of China (82171305, 81971134 and 82001276); Xuzhou Innovation Capacity Building Program (grant no. KC19239); The Natural Science Foundation of Jiangsu Province (BK20191152); Medical Scientific Research Project of Jiangsu Provincial Health Commission (ZDB2020017); Xuzhou Key Research and Development Program (KC19131); Postgraduate Research&Practice Innovation Program of Jiangsu Province (KYCX22‐2947).

## CONFLICT OF INTEREST STATEMENT

The authors declare no competing interests.

### PEER REVIEW

The peer review history for this article is available at https://www.webofscience.com/api/gateway/wos/peer-review/10.1002/btm2.10590.

## Supporting information


**Data S1.** Supporting Information.

## Data Availability

The data used in the study are available from the corresponding author on reasonable request.

## References

[1] Feigin V , Roth G , Naghavi M , et al. Global burden of stroke and risk factors in 188 countries, during 1990–2013: a systematic analysis for the global burden of disease study 2013. Lancet Neurol. 2016;15(9):913‐924. 27291521 10.1016/S1474-4422(16)30073-4

[2] Walter K . What is acute ischemic stroke? JAMA. 2022;327(9):885. 35230392 10.1001/jama.2022.1420

[3] Jovin T , Nogueira R , Lansberg M , et al. Thrombectomy for anterior circulation stroke beyond 6 h from time last known well (AURORA): a systematic review and individual patient data meta‐analysis. Lancet (London, England). 2022;399(10321):249‐258. 34774198 10.1016/S0140-6736(21)01341-6

[4] Chen G , Ye X , Zhang J , et al. Limb remote ischemic postconditioning reduces ischemia–reperfusion injury by inhibiting NADPH oxidase activation and MyD88‐TRAF6‐P38MAP‐kinase pathway of neutrophils. Int J Mol Sci. 2016;17(12):1971. 10.3390/ijms17121971 PMC5187771 27898007

[5] Zhao H , Ren C , Chen X , Shen J . From rapid to delayed and remote postconditioning: the evolving concept of ischemic postconditioning in brain ischemia. Curr Drug Targets. 2012;13(2):173‐187. 22204317 10.2174/138945012799201621 PMC3346695

[6] Guo L , Zhou D , Wu D , et al. Short‐term remote ischemic conditioning may protect monkeys after ischemic stroke. Ann Clin Transl Neurol. 2019;6(2):310‐323. 30847363 10.1002/acn3.705 PMC6389742

[7] Vaibhav K , Braun M , Khan M , et al. Remote ischemic post‐conditioning promotes hematoma resolution via AMPK‐dependent immune regulation. J Exp Med. 2018;215(10):2636‐2654. 30190288 10.1084/jem.20171905 PMC6170180

[8] Vinciguerra A , Cepparulo P , Anzilotti S , et al. Remote postconditioning ameliorates stroke damage by preventing let‐7a and miR‐143 up‐regulation. Theranostics. 2020;10(26):12174‐12188. 33204336 10.7150/thno.48135 PMC7667695

[9] Han D , Wang J , Wen L , et al. Remote limb ischemic postconditioning protects against ischemic stroke via modulating microglia/macrophage polarization in mice. J Immunol Res. 2021;2021:6688053. 33688509 10.1155/2021/6688053 PMC7910075

[10] Iadecola C , Anrather J . The immunology of stroke: from mechanisms to translation. Nat Med. 2011;17(7):796‐808. 21738161 10.1038/nm.2399 PMC3137275

[11] Sarhan M , Land W , Tonnus W , et al. Origin and consequences of necroinflammation. Physiol Rev. 2018;98(2):727‐780. 29465288 10.1152/physrev.00041.2016

[12] Jiang W , Li M , He F , Zhou S , Zhu L . Targeting the NLRP3 inflammasome to attenuate spinal cord injury in mice. J Neuroinflammation. 2017;14(1):207. 29070054 10.1186/s12974-017-0980-9 PMC5657095

[13] Liu W , Chen Y , Meng J , et al. Ablation of caspase‐1 protects against TBI‐induced pyroptosis in vitro and in vivo. J Neuroinflammation. 2018;15(1):48. 29458437 10.1186/s12974-018-1083-y PMC5817788

[14] Zhang D , Qian J , Zhang P , et al. Gasdermin D serves as a key executioner of pyroptosis in experimental cerebral ischemia and reperfusion model both in vivo and in vitro. J Neurosci Res. 2019;97(6):645‐660. 30600840 10.1002/jnr.24385

[15] Mamik M , Power C . Inflammasomes in neurological diseases: emerging pathogenic and therapeutic concepts. Brain J Neurol. 2017;140(9):2273‐2285. 10.1093/brain/awx133 29050380

[16] Lamkanfi M , Dixit V . Mechanisms and functions of inflammasomes. Cell. 2014;157(5):1013‐1022. 24855941 10.1016/j.cell.2014.04.007

[17] Shi J , Gao W , Shao F . Pyroptosis: gasdermin‐mediated programmed necrotic cell death. Trends Biochem Sci. 2017;42(4):245‐254. 27932073 10.1016/j.tibs.2016.10.004

[18] Matsumoto K , Nakamura T . Emerging multipotent aspects of hepatocyte growth factor. J Biochem. 1996;119(4):591‐600. 8743556 10.1093/oxfordjournals.jbchem.a021283

[19] Kitamura K , Iwanami A , Nakamura M , et al. Hepatocyte growth factor promotes endogenous repair and functional recovery after spinal cord injury. J Neurosci Res. 2007;85(11):2332‐2342. 17549731 10.1002/jnr.21372

[20] Sowa K , Nito C , Nakajima M , et al. Impact of dental pulp stem cells overexpressing hepatocyte growth factor after cerebral ischemia/reperfusion in rats. Mol Ther Methods Clin Dev. 2018;10:281‐290. 30151417 10.1016/j.omtm.2018.07.009 PMC6108066

[21] Nishikoba N , Kumagai K , Kanmura S , et al. HGF‐MET signaling shifts M1 macrophages toward an M2‐like phenotype through PI3K‐mediated induction of Arginase‐1 expression. Front Immunol. 2020;11:2135. 32983173 10.3389/fimmu.2020.02135 PMC7492554

[22] Peng F , Chang W , Sun Q , et al. HGF alleviates septic endothelial injury by inhibiting pyroptosis via the mTOR signalling pathway. Respir Res. 2020;21(1):215. 32795289 10.1186/s12931-020-01480-3 PMC7427898

[23] Farrell J , Kelly C , Rauch J , et al. HGF induces epithelial‐to‐mesenchymal transition by modulating the mammalian hippo/MST2 and ISG15 pathways. J Proteome Res. 2014;13(6):2874‐2886. 24766643 10.1021/pr5000285

[24] Zhang X , Bogunovic D , Payelle‐Brogard B , et al. Human intracellular ISG15 prevents interferon‐α/β over‐amplification and auto‐inflammation. Nature. 2015;517(7532):89‐93. 25307056 10.1038/nature13801 PMC4303590

[25] Xu P , Hong Y , Xie Y , et al. TREM‐1 exacerbates neuroinflammatory injury via NLRP3 inflammasome‐mediated Pyroptosis in experimental subarachnoid hemorrhage. Transl Stroke Res. 2021;12(4):643‐659. 32862402 10.1007/s12975-020-00840-x

[26] Xu P , Zhang X , Liu Q , et al. Microglial TREM‐1 receptor mediates neuroinflammatory injury via interaction with SYK in experimental ischemic stroke. Cell Death Dis. 2019;10(8):555. 31324751 10.1038/s41419-019-1777-9 PMC6642102

[27] Bergsbaken T , Fink S , Cookson B . Pyroptosis: host cell death and inflammation. Nat Rev Microbiol. 2009;7(2):99‐109. 19148178 10.1038/nrmicro2070 PMC2910423

[28] Ren C , Yan Z , Wei D , Gao X , Chen X , Zhao H . Limb remote ischemic postconditioning protects against focal ischemia in rats. Brain Res. 2009;1288:88‐94. 19631625 10.1016/j.brainres.2009.07.029 PMC2744502

[29] Valsecchi V , Laudati G , Cuomo O , et al. The hypoxia sensitive metal transcription factor MTF‐1 activates NCX1 brain promoter and participates in remote postconditioning neuroprotection in stroke. Cell Death Dis. 2021;12(5):423. 33931586 10.1038/s41419-021-03705-9 PMC8087832

[30] Xu W , Jin W , Zhang X , Chen J , Ren C . Remote limb preconditioning generates a neuroprotective effect by modulating the extrinsic apoptotic pathway and TRAIL‐receptors expression. Cell Mol Neurobiol. 2017;37(1):169‐182. 26971954 10.1007/s10571-016-0360-5 PMC11482232

[31] Yang J , Balkaya M , Beltran C , et al. Remote Postischemic conditioning promotes stroke recovery by shifting circulating monocytes to CCR2 proinflammatory subset. J Neurosci. 2019;39(39):7778‐7789. 31427395 10.1523/JNEUROSCI.2699-18.2019 PMC6764204

[32] Guo Z , Guo W , Liu J , et al. Changes in cerebral autoregulation and blood biomarkers after remote ischemic preconditioning. Neurology. 2019;93(1):e8‐e19. 31142636 10.1212/WNL.0000000000007732 PMC6659004

[33] Próchnicki T , Mangan M , Latz E . Recent insights into the molecular mechanisms of the NLRP3 inflammasome activation. F1000Research. 2016;5:5. 10.12688/f1000research.8614.1 PMC4963208 27508077

[34] McKenzie B , Fernandes J , Doan M , et al. Activation of the executioner caspases‐3 and ‐7 promotes microglial pyroptosis in models of multiple sclerosis. J Neuroinflammation. 2020;17(1):253. 32861242 10.1186/s12974-020-01902-5 PMC7456507

[35] Xue Y , Enosi Tuipulotu D , Tan W , et al. Emerging activators and regulators of inflammasomes and Pyroptosis. Trends Immunol. 2019;40(11):1035‐1052. 31662274 10.1016/j.it.2019.09.005

[36] Hu J , Ma W , He L , et al. Macrophage migration inhibitory factor (MIF) acetylation protects neurons from ischemic injury. Cell Death Dis. 2022;13(5):466. 35585040 10.1038/s41419-022-04918-2 PMC9117661

[37] Walsh J , Muruve D , Power C . Inflammasomes in the CNS. Nat Rev Neurosci. 2014;15(2):84‐97. 24399084 10.1038/nrn3638

[38] Luo Y , Reis C , Chen S . NLRP3 inflammasome in the pathophysiology of hemorrhagic stroke: a review. Curr Neuropharmacol. 2019;17(7):582‐589. 30592254 10.2174/1570159X17666181227170053 PMC6712291

[39] Wu X , Wan T , Gao X , et al. Microglia pyroptosis: a candidate target for neurological diseases treatment. Front Neurosci. 2022;16:922331. 35937897 10.3389/fnins.2022.922331 PMC9354884

[40] Zalpoor H , Akbari A , Samei A , et al. The roles of Eph receptors, neuropilin‐1, P2X7, and CD147 in COVID‐19‐associated neurodegenerative diseases: inflammasome and JaK inhibitors as potential promising therapies. Cell Mol Biol Lett. 2022;27(1):10. 35109786 10.1186/s11658-022-00311-1 PMC8809072

[41] Cyr B , Hadad R , Keane R , et al. The role of non‐canonical and canonical inflammasomes in Inflammaging. Front Mol Neurosci. 2022;15:774014. 35221912 10.3389/fnmol.2022.774014 PMC8864077

[42] Groslambert M , Py B . Spotlight on the NLRP3 inflammasome pathway. J Inflamm Res. 2018;11:359‐374. 30288079 10.2147/JIR.S141220 PMC6161739

[43] Global, regional, and national burden of neurological disorders, 1990–2016: a systematic analysis for the global burden of disease study 2016. Lancet Neurol. 2019;18(5):459‐480. 30879893 10.1016/S1474-4422(18)30499-X PMC6459001

[44] Albornoz E , Woodruff T , Gordon R . Inflammasomes in CNS diseases. Exp Suppl. 2012;2018(108):41‐60. 10.1007/978-3-319-89390-7_3 30536167

[45] Hess D , Blauenfeldt R , Andersen G , et al. Remote ischaemic conditioning‐a new paradigm of self‐protection in the brain. Nat Rev Neurol. 2015;11(12):698‐710. 26585977 10.1038/nrneurol.2015.223

[46] Liu M , Dziennis S , Hurn P , et al. Mechanisms of gender‐linked ischemic brain injury. Restor Neurol Neurosci. 2009;27(3):163‐179. 19531872 10.3233/RNN-2009-0467 PMC2826890

[47] Ye X , Shen T , Hu J , et al. Purinergic 2X7 receptor/NLRP3 pathway triggers neuronal apoptosis after ischemic stroke in the mouse. Exp Neurol. 2017;292:46‐55. 28274860 10.1016/j.expneurol.2017.03.002

[48] Monai H , Wang X , Yahagi K , et al. Adrenergic receptor antagonism induces neuroprotection and facilitates recovery from acute ischemic stroke. Proc Natl Acad Sci U S A. 2019;116(22):11010‐11019. 31097598 10.1073/pnas.1817347116 PMC6561179

[49] Wu Y , Yang L , Mei X , Yu Y . Selective inhibition of STAT1 reduces spinal cord injury in mice. Neurosci Lett. 2014;580:7‐11. 24321405 10.1016/j.neulet.2013.11.055

[50] Schmittgen T , Livak K . Analyzing real‐time PCR data by the comparative C(T) method. Nat Protoc. 2008;3(6):1101‐1108. 18546601 10.1038/nprot.2008.73

[51] Ye X , Hao Q , Ma W , et al. Dectin‐1/Syk signaling triggers neuroinflammation after ischemic stroke in mice. J Neuroinflammation. 2020;17(1):17. 31926564 10.1186/s12974-019-1693-z PMC6954534

