# Supplementary material for: Remote limb ischemic postconditioning inhibits microglia pyroptosis by modulating HGF after acute ischemia stroke
Source: Bioeng Transl Med. 2023 Aug 19;8(6):e10590. doi: 10.1002/btm2.10590 (PMC10658568; doi:10.1002/btm2.10590)
Supplement: Supplementary file 1 — Data S1. Supporting Information. [file BTM2-8-e10590-s001.docx]

**SUPPLEMENTAL MATERIAL**

**Remote Limb Ischemic Postconditioning Inhibits Microglia Pyroptosis by Modulating HGF After Acute Ischemia Stroke**

**Supplemental Tables**

**Table S1. Summary of experimental groups and mortality rate in the study**

| **Experimental Groups** | **Behavior assessment** | **TTC**  **rCBF** | **WB**  **ELISA**  **HTC**  **PCR** | **IF**  **IHC**  **EM** | **Exclusion** | **Mortality**  **(%)** | **Subtotal** |
| --- | --- | --- | --- | --- | --- | --- | --- |
| **Experimental 1** |  |  |  |  |  |  |  |
| Sham |  | 3 | 16 | 3 | 0 | 0 | 22 |
| MCAO | 9 | 6 | 16 | 4 | 0 | 5(12.5) | 40 |
| MCAO+RIPostC | 9 | 6 | 16 | 4 | 0 | 4(7.89) | 39 |
| **Experimental 2** |  |  |  |  |  |  |  |
| MCAO+Con SiRNA |  |  | 3 |  | 0 | 1(25) | 4 |
| MCAO+HGF SiRNA |  |  | 3 |  | 0 | 0 | 3 |
| MCAO+RIPostC+Con SiRNA |  |  | 3 |  | 0 | 0 | 3 |
| MCAO+RIPostC+HGF SiRNA |  |  | 3 |  | 0 | 0 | 3 |
| MCAO+Con SiRNA |  |  | 3 |  | 0 | 0 | 3 |
| MCAO+ISG15 SiRNA |  |  | 3 |  | 0 | 0 | 3 |
| MCAO+RIPostC+Con SiRNA |  |  | 3 |  | 0 | 0 | 3 |
| MCAO+RIPostC+ISG15 SiRNA |  |  | 3 |  | 0 | 1 | 4 |
| **Experimental 3** |  |  |  |  |  |  |  |
| Sham |  |  | 8 | 7 | 0 |  | 15 |
| MCAO+Con SiRNA |  |  | 8 | 7 | 0 | 2(11.76) | 17 |
| MCAO+RIPostC+Con SiRNA |  |  | 8 | 7 | 0 | 2(11.76) | 17 |
| MCAO+RIPostC+HGF SiRNA |  |  | 8 | 7 | 0 | 2(11.76) | 17 |
| MCAO+RIPostC+ISG15 SiRNA |  |  | 8 | 7 | 0 | 3(16.67) | 18 |
| **Total** | 18 | 15 | 112 | 46 | 0 | 20(9.47) | 211 |

**Table S2 Power analysis**

| **Group1** | **Group2** | **Group3** | **Group4** | **Group5 n** | | |  | **Power** | |
| --- | --- | --- | --- | --- | --- | --- | --- | --- | --- |
| **Sham** | **MCAO** | **MCAO+RIPostC** | - | - | 3 | 0.986 | | |  |
| 7634.72644 | 2881.87605 | 5473.73177 | - | - |  |  |  |  |  |
| 7979.78055 | 3279.43717 | 6423.87600 | - | - |  |  |  |  |  |
| 8077.53590 | 3264.26738 | 4502.92750 | - | - |  |  |  |  |  |
| **MCAO+**  **ConSiRNA** | **MCAO+ISG15 SiRNA** | **MCAO+RIPostC+Con SiRNA** | **MCAO+RIPostC+ISG15 SiRNA** | - | 3 | 0.997 | | |  |
| 0.3865916 | 0.2049893 | 0.6219754 | 0.2312328 | - |  |  |  |  |  |
| 0.5128066 | 0.2706690 | 0.5710934 | 0.4204448 | - |  |  |  |  |  |
| 0.4334154 | 0.3367340 | 0.5679397 | 0.3731600 | - |  |  |  |  |  |
| **Sham** | **MCAO+Con SiRNA** | **MCAO+RPC+Con SiRNA** | **MCAO+RPC+HGF SiRNA** | **MCAO+RPC+ISG15 SiRNA** |  |  | | |  |
| 0.3074795 | 0.4628019 | 0.2689849 | 0.3822054 | 0.378463 | 3 | 0.86 | | |  |
| 0.2877733 | 0.4163291 | 0.2734588 | 0.4205121 | 0.4528276 |  |  | | |  |
| 0.2695689 | 0.334722 | 0.2427720 | 0.3707175 | 0.3540949 |  |  | | |  |

**Supplemental Figures with Figure Legends**

Figure S1:

Figure S1:tthe analysis of GSDMD positive microglia in ischemic penumbra. Data presented as mean ± SEM. *P < 0.05; **P < 0.01; ***P < 0.001.

Figure S2:


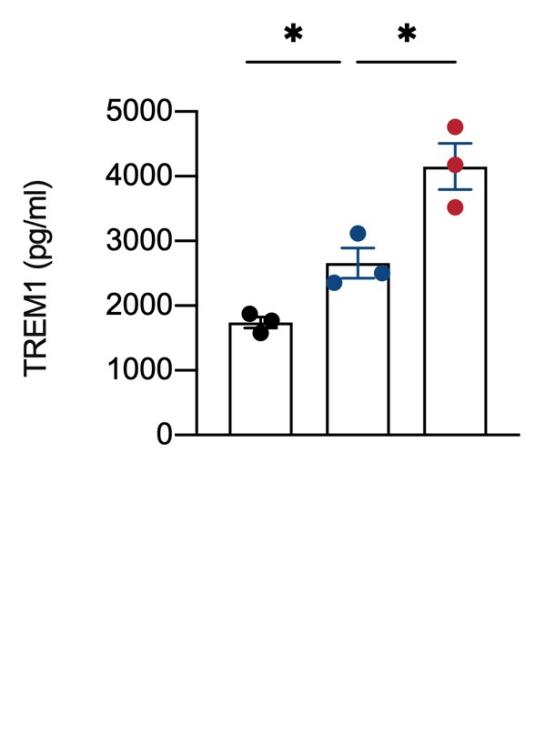

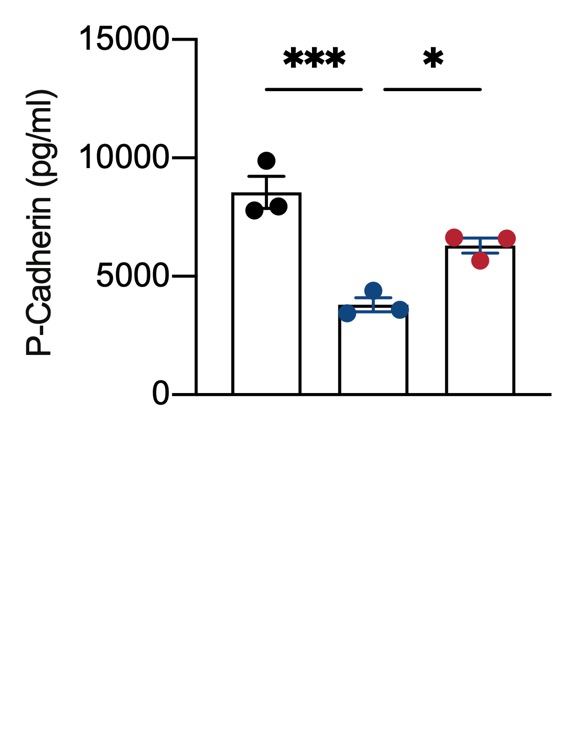


Figure S2:the analysis of TREM1 and P-Cadherin expression in protein chip. Data presented as mean ± SEM. *P < 0.05; **P < 0.01; ***P < 0.001.

Figure S3:


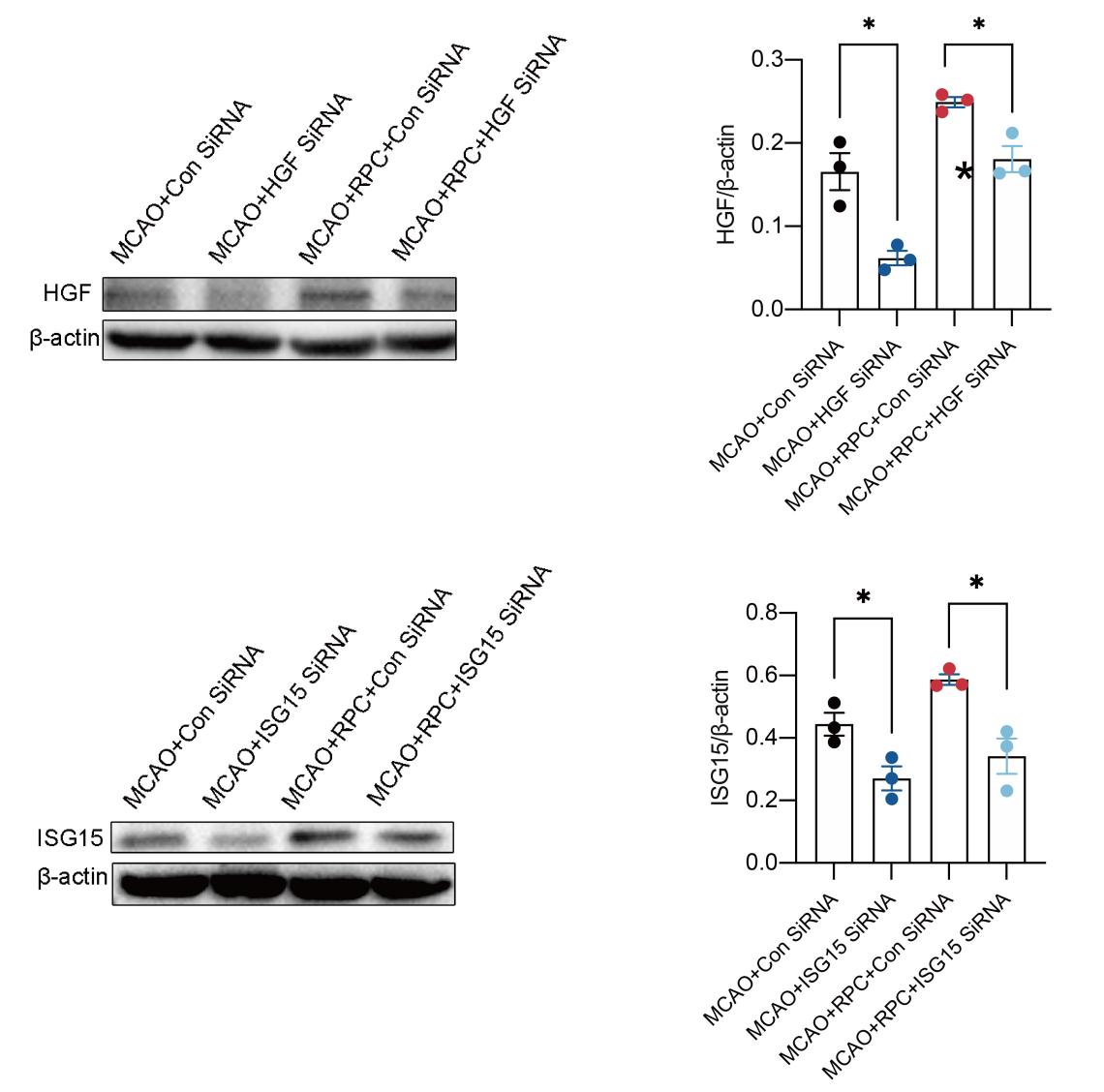


Figure S3: Representative western blot bands and quantification of HGF or ISG15 knockdown efficacy in MCAO mice with or without RIPostC treatment at 3 day after i.c.v. injection.

Figure S4


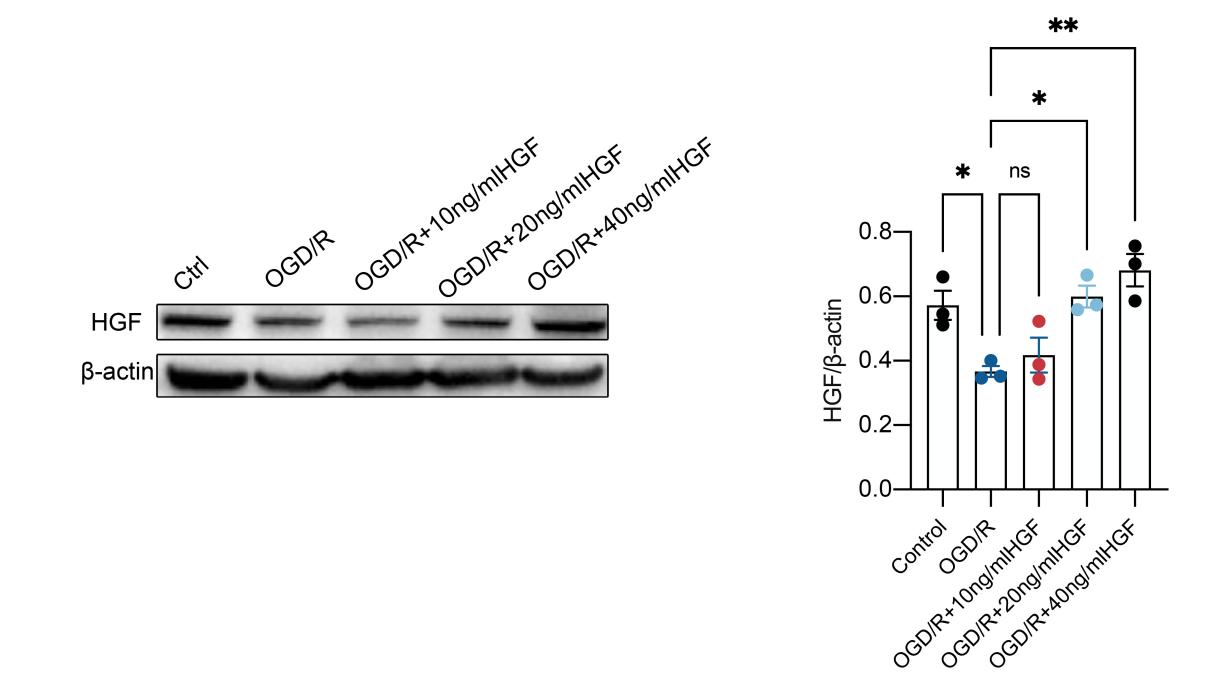


Figure S4: The HGF protein expression in exogenous HGF-treated BV2 cells under OGD/R conditions. n=3. ∗P < 0.05.

Figure S5


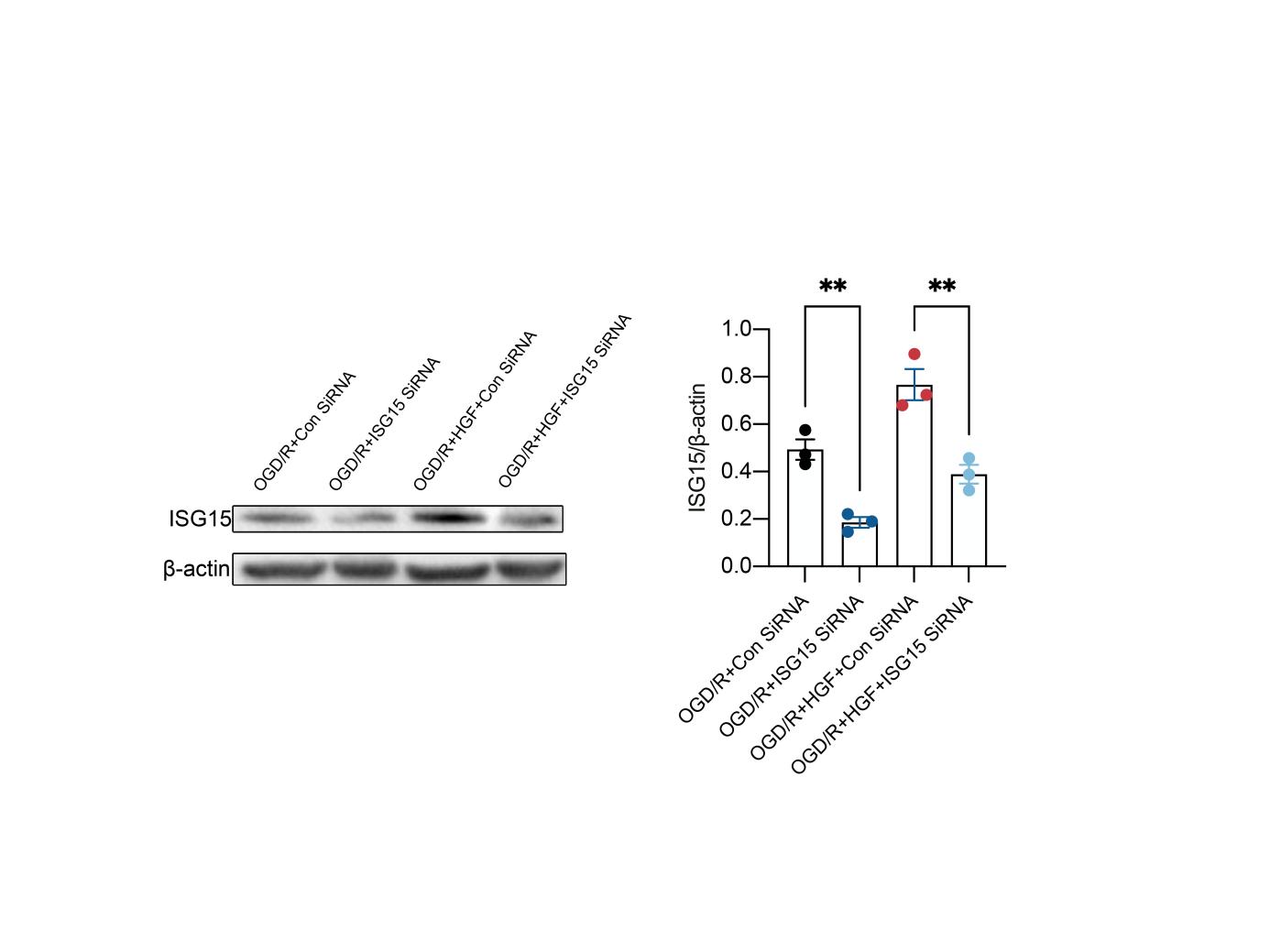


Figure S5:ISG15 protein expression in different treated groups. n=3.*P < 0.05; **P < 0.01; ***P < 0.001.
